# Effect of Different Species of *Prorocentrum* Genus on the Japanese Oyster *Crassostrea gigas* Proteomic Profile

**DOI:** 10.3390/toxins13070504

**Published:** 2021-07-20

**Authors:** Miguel Angel Matus Hernández, Norma Yolanda Hernández Saavedra

**Affiliations:** Centro de Investigaciones Biológicas del Noroeste, S.C. (CIBNOR), Av. Instituto Politécnico Nacional No. 195, Col. Playa Palo de Sta. Rita, P.O. Box 128, La Paz, Baja California Sur 23096, Mexico; matus.hernandez.miguel@gmail.com

**Keywords:** *Prorocentrum*, *Crassostrea gigas*, proteomic maps, toxin effects, in vitro exposure

## Abstract

This paper assesses the effects of exposure to toxic concentrations (1200 to 6000 cells/mL) of the dinoflagellates *Prorocentrum lima*, *Prorocentrum minimum*, and *Prorocentrum rhathymum* and several concentrations of aqueous and organic extracts obtained from the same species (0 to 20 parts per thousand) on the *Crassostrea gigas* (5–7 mm) proteomic profile. Through comparative proteomic map analyses, several protein spots were detected with different expression levels, of which eight were selected to be identified by liquid chromatography–mass spectrometry (LC–MS/MS) analyses. The proteomic response suggests that, after 72 h of exposure to whole cells, the biological functions of *C. gigas* affected proteins in the immune system, stress response, contractile systems and cytoskeletal activities. The exposure to organic and aqueous extracts mainly showed effects on protein expressions in muscle contraction and cytoskeleton morphology. These results enrich the knowledge on early bivalve developmental stages. Therefore, they may be considered a solid base for new bioassays and/or generation of specific analytical tools that allow for some of the main effects of algal proliferation phenomena on bivalve mollusk development to be monitored, characterized and elucidated.

## 1. Introduction

Phycotoxins are natural metabolites produced by micro-algae or seaweeds, which are mass molecules of around 300–3500 Da that belong to diverse groups of chemical compounds [1,2]. In the marine environment, these organic compounds are produced by diatoms, dinoflagellates, cyanobacteria, and other flagellated phytoplankton [1,3]. Of the 5000 phytoplankton species known, about 300 of them are involved in proliferation events [4,5]. These events are natural phenomena characterized by an exponential increase in cell density, as a result of changes in several environmental factors (temperature, salinity, nutrients, ocean acidification, precipitation, etc.). Nevertheless, how the integration of these climate drivers might have driven proliferation is still unclear [6].

Among the 300 phytoplankton species mentioned above, only about 100 produce toxins that can cause intoxication or even death in humans and animals [5]. The phytoplankton species mostly involved in these toxic events are dinoflagellates and diatoms. Furthermore, species of the genus *Prorocentrum* have been recurrently reported from tropical and temperate waters. The genus *Prorocentrum* is a group of dinoflagellates distributed worldwide in planktonic and benthic marine ecosystems, with 78 species accepted taxonomically hitherto [7]. At least ten species of *Prorocentrum* (*P. lima*, *P. cordatum* (as *P. minimum*), *P. borbonicum*, *P. concavum*, *P. leve*, *P. rhathymum*, *P. hoffmannianum* (as *P. maculosum*), *P. caipirignum*, *P. belizeanum*, and *P. faustiae*) have been confirmed to produce a suite of toxins called okadaic acid (OA) and its analogues, also referred to as dinophysistoxins (DTXs). Additionally, borbotoxins and other unidentified toxins have been associated with diarrheic shellfish poisoning [8,9]. These compounds are complex lipid- and water-soluble polyether molecules and are specific inhibitors of serine/threonine phosphatases at the molecular level. However, at cellular levels, they cause alterations in DNA and on cellular components as well as effects on immune and nervous systems and on embryonic development [10]. 

Phycotoxins are acquired by fish and shellfish—mainly bivalve mollusks (oysters, mussels, scallops, or clams)—by direct filtration of dinoflagellates from the water column and via feeding on resuspended benthic material. Subsequently, they can be consumed by humans, causing alimentary intoxication. Studies on the effects of the genus *Prorocentrum* on bivalve mollusks have been performed, but most of them have addressed biological responses, such as particle selection, survival, clearance rate, depuration, growth, and toxin accumulation [11,12,13,14,15,16,17]. Moreover, studies at the molecular level are scarce despite the wide interest in knowing the cellular response mechanisms caused by organisms of the genus *Prorocentrum* [18,19,20,21,22,23].

The Pacific oyster *C. gigas* is an ideal marine invertebrate model for the studies previously mentioned because of its ecological and economic significance [24]. In addition to its biology, genetics, and innate immunity being extensively studied [25,26], it is the first marine sessile bivalve for which the genome has been completely sequenced [27]. *C. gigas* inhabits estuarine and intertidal zones, where it is exposed to dramatic environmental fluctuations, including phytoplankton proliferations. Thus, vast interest exists in understanding several cellular response mechanisms to alterations in the physical-chemical environment at the organism level. Proteome rearrangements are common responses to environmental stress, so they must be distinctive of certain types of toxic exposure [28]. Currently, proteomic studies conducted on bivalves have been focused primarily on protein expression profile analyses after exposure to a variety of environmental contaminants [29,30,31,32,33,34,35,36,37]. Others have investigated the response to ocean acidification [38,39,40,41] and evaluated the effect of toxic phytoplankton [42]. Some studies have revealed that early stages of development are the most susceptible to stress and that >7% of the oyster larval proteome is altered, affecting growth, development, and fitness and causing shell morphological abnormalities in these juvenile oysters. Therefore, this study investigates the effects of three toxic *Prorocentrum* species (*P. lima*, *P. minimum*, and *P. rhathymum*) and their aqueous (AE) and organic (OE) extracts on *C. gigas* spat proteomic response.

## 2. Results

### 2.1. Mortality of C. gigas Exposed to Prorocentrum Complete Cells or Extracts (Time, Number, and Concentration)

**Figure 1:**
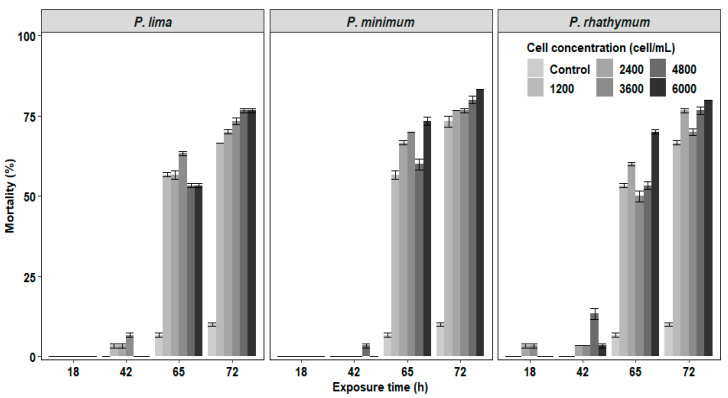
Percentage of cumulative mortality of *C. gigas* oyster spat exposed throughout time to several concentrations of whole cells (1200 2400 3600 4800 and 6000 cells/mL) of three different *Prorocentrum* species. Note: *Isochrysis galbana* at 6000 cells/mL^−1^ was used as the control group.

**Figure 2:**
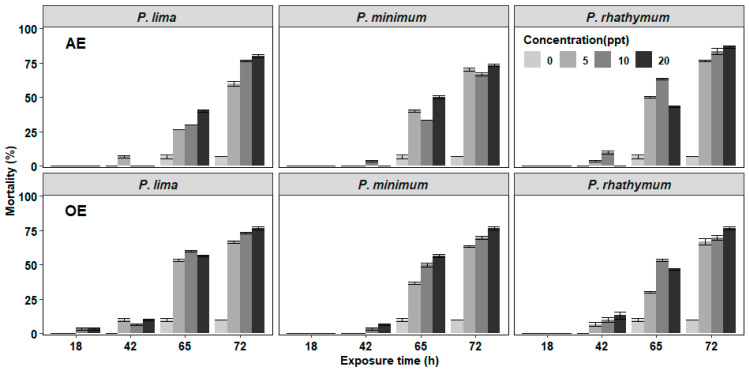
Percentage of cumulative mortality of *C. gigas* oyster spat exposed throughout time to several concentrations of aqueous or organic extracts (AE and OE, respectively) of three different *Prorocentrum* species. Note: Extract concentrations used: 0, 5, 10, and 20 parts per thousand (ppt). As controls, distilled water and 1% Tween 60 were used on AE and OE experiments (respectively).

In the experiments of *C. gigas* with exposure to complete cells or extracts of *Prorocentrum* species, the mortality of juveniles was observed after 42 h exposure with the highest number of deaths at 72 h without reaching 100% at the end of the bioassay (Figure 1 and Figure 2). In organisms exposed to whole cells (Figure 1), the highest cumulative mortality was observed with treatments of 6000 cells/mL; *P. minimun* caused the highest mortality (83.3%), followed by *P. rhathymum* (80%) and *P. lima* (76.6%). Similarly, organisms exposed to different extracts showed massive mortality after 42 h exposure (Figure 2). The highest cumulative mortality occurred with extract concentrations of 20 parts per thousand (ppt). As in the case of exposure to whole cells, the cumulative mortality using AE varied according to the *Prorocentrum* species. The highest cumulative mortality was observed with *P. rhathymum* (86.6%), followed by *P. lima* (80%) and *P. minimum* (73.3%). With OE, the three *Prorocentrum* species promoted similar cumulative mortality percentages in *C. gigas* (76.6%), while in the control group, mortality reached only 6.67%.

When the effects caused by each considered factor (number of cells/mL^−1^, species, exposure time) on *C. gigas* mortality were compared in each experiment (Table 1), the statistical analyses showed: (1) whole cell exposure (Table S1), in which (i) cell concentration of *Prorocentrum* species did not influence spat mortality significantly (*p* < 0.05) and (ii) significant differences were observed on mortality percentage between *P. minimum* with respect to *P. lima* and *P. rhathymum* after 65 h of exposure (*p* < 0.05); (2) aqueous (AE) or organic (OE) extract cell exposure. Similar results were observed when any *Prorocentrum* species was used: (i) No significant differences (*p* < 0.05) were observed between the tested concentrations of both extracts (AE or OE); thus, the tested extract concentration is not a factor that affects mortality. However, significant differences on *C. gigas* mortality were observed (*p* < 0.05) only after 65 and 72 h exposure to *Prorocentrum* species extracts. (ii) When AE was used, statistically significant differences were observed only on *C. gigas* mortality exposed to *Prorocentrum* species with regard to those of the control group (*p* < 0.05) (Table S2). (iii) When OE was tested, no significant differences were observed in oyster mortality between organisms exposed to *P. lima*, *P. minimum* and *P. rhathymum* with regard to oysters exposed to the control treatment (*p* < 0.05) (Table S3).

### 2.2. Proteomic Response

**Figure 3:**
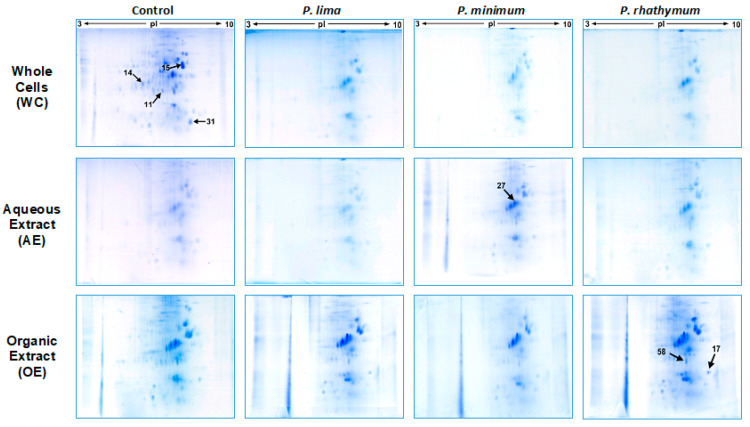
Representative changes on 2D-GE protein patterns of *C. gigas* spat in response to exposure to whole cells (WC), aqueous (AE), and organic (OE) extracts of *P. lima*, *P. minimum*, or *P. rhathymum*; *I. galbana* was used as the control. For whole cell assays, 6000 cells/mL of each species were used. For exposure to extract assays, 20 parts per thousand (ppt) were used on each case, and AE = Distilled water and OE = 1% Tween 60 were used as controls.

Considering the results obtained in the mortality assay, organisms exposed to 6000 cells/mL and extract concentrations of 20 ppt from each dinoflagellate species were used for the proteomic analyses. In this manner, 12 protein extracts were prepared by homogenization of 20 spat for each treatment (in triplicate), obtaining samples with around 3 mg/mL of total soluble proteins (in average); proteomic maps (2D) of soluble proteins from all assays are shown in Figure 3. In all cases, 2D maps were similar among triplicates showing a high reproducibility of two-dimensional gel electrophoresis (2D-GE) technique in this study. 

**Figure 4:**
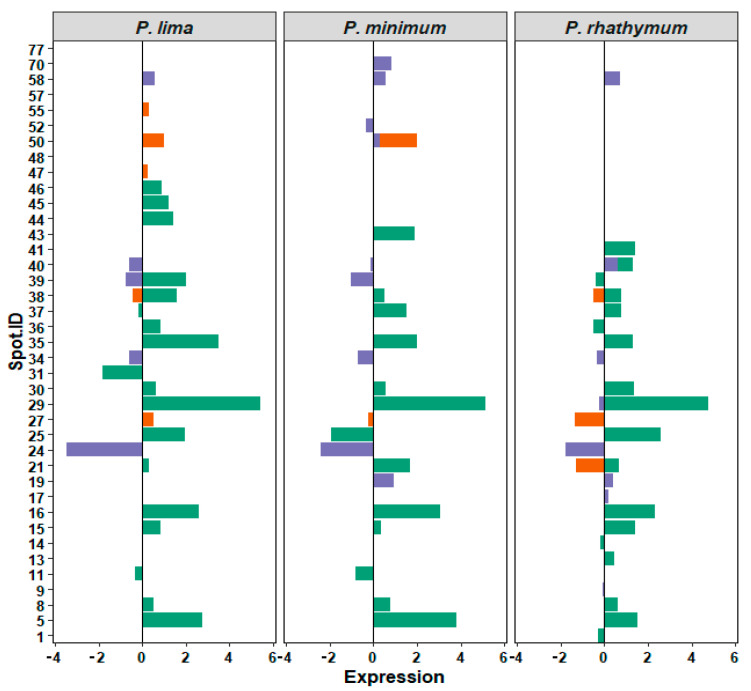
Relative expression of 39 regulated protein spots identified by 2D-GE image analyses. Bars corresponds to treatments (green WC, whole cells; orange AE, aqueous extract; and violet OE, organic extract). X axis: relative expression on arbitrary units. Zero means no changes with respect to control, and signs, positive or negative, denotes overexpression or subexpression, respectively. Y axis, ID (identification number) of each spot. Panels correspond to species: *P. lima*, *P. minimum*, or *P. rhathymum*.

Analyzing 2D-GE images of the treatment groups with respect to the control, a pattern of 434 protein spots were observed in treatments with whole cells, distributed in a pH range from 5 to 8 with mass (Mr) values from 200 to 20 kDa. From these spots (Table 2), the statistical analysis revealed significant differences (*p* < 0.05) in intensity levels of 26 protein spots when the control was compared with each *Prorocentrum* species (Figure 3, Table S4). When oysters were exposed to *P. lima*, upregulation of 14 protein spots as well as downregulation, suppression, and induction of three protein spots for each case were observed. *P. minimum* cells promoted the upregulation of ten protein spots, the downregulation and suppression of five protein spots, and the induction of one protein spot. Finally, *C. gigas* exposure to *P. rhathymum* cells promoted the upregulation of 13 protein spots, the downregulation of four spots, the suppression of three spots, and the induction of two (Table 2 and Figure 4).

In the case of oysters exposed to AE (20 ppt), the analyses of 2D-GE images showed a pattern of 476 protein spots distributed in a pH range from 6 to 8 (Figure 3). From the 476 spots, the statistical analysis revealed significant differences (*p* < 0.05) in seven spots (Figure 4 and Table S4). Under these exposure conditions, the suppressed number of protein spots was higher than any other exposure condition, mainly in extract presence of *P. minimum* (4 spots), followed by *P. rhathymum* and *P. lima* (two proteins spots, respectively). Downregulation was observed on the protein 2D-GE patterns of organisms exposed to *P. rhathymum* and *P. lima* extracts (three and one protein spots, respectively), while upregulated proteins were observed with *P. lima* (two) and *P. minimum* (one). Finally, induced proteins appeared only in extracts from oysters exposed to *P. lima* (two) and *P. minimum* (one) (Table 2 and Figure 4).

In the case of OE (20 ppt), the comparison between 2D-GE protein patterns showed that *C. gigas* obtained from the control and extract treatments revealed a pattern of 623 protein spots, distributed in a pH range from 6 to 8 (Figure 3). The statistical analysis revealed significant differences (*p* < 0.05) in 13 of the 623 protein spots (Figure 4 and Table S4). In this experiment, the highest number of downregulated protein spots was observed in the presence of *P. minimum* extracts (six), followed by *P. rhathymum* and *P. lima* (four for each one). The upregulation of three protein spots was observed when extracts of *P. rhathymum* or *P. minimum* were used, and only one with *P. lima.* The suppressed proteins were six with the *P. lima* extract and three with *P. rhathymum* and *P. minimum*. Finally, the induced proteins were two with *P. minimum*, and one each with *P. lima* or *P. rhathymum* species (Table 2 and Figure 4).

### 2.3. Protein Identification

A total of seven isolated protein spots were successfully identified, four from assays of *C. gigas* exposure to whole cells of three *Prorocentrum* species, one from exposure to AE, and two from assays of exposure to OE (Table 3 and Table S5). Multiple proteins were identified in some spots: three in spots 14 and 58 and two in spots 11 and 27.

## 3. Discussion

Currently, a wide variety of chemical, biological, and molecular techniques and methods have been developed that can be used efficiently for the study of marine toxins; nevertheless, their toxic potential can only be adequately assessed by bioassays with living organisms, which provide a measure of the total toxicity based on the organisms’ biological response to toxins [43]. Bioassays with bivalves have been commonly used in recent decades to determine the physiological and behavioral exposure responses to organisms producing algal proliferation [11,12]. The direct exposure of bivalves to whole cells of algal proliferating organisms is one of the most widely used bioassays to assess toxicity effect. As cells enter the mollusks through a natural feeding process, they represent the natural exposure scenario, and the observations obtained reflect the effects caused by an algal proliferation scenario more reliably.

In regard to oyster mortality, no significant differences among treatments and controls were observed. In the bioassay with whole cells, the results suggest that cell number does not influence mortality on each species. However, when the results were compared among *Prorocentrum* species, significant differences were observed between *P. minimum* regarding *P. lima* and *P. rhathymum* (Figure 1 and Figure 2). These results could be explained by the ability of mollusks to classify and select their food, where the main characteristics involved are particle size and shape. In addition to their chemical and/or biochemical characteristics, size and shape played a main role in this research study because *C. gigas* spat fed mainly small cells of phytoplankton, while larger cells had lower or moderate ingestion rates [44]. The cell size used in this research was 18–20 μm long and 15–16 μm wide in *P. minimum*, 32–50 μm long and 20–28 μm wide in *P. lima* [45], and 31–36 µm long and 17–21 µm wide in *P. rhathymum* [46].

Likewise, this ability to classify and select food could be one of the causes of pseudo-feces production observed, especially in organisms fed with *P. lima* and *P. rhathymum* where larger cells were rejected by the gills and lip palps, or covered with mucus to later be removed from the inhalation chamber in the form of pseudo-feces. These results were consistent with previous studies carried out in juveniles of *C. gigas* where exposure to *P. lima* cells [47,48] as well as to a mixture of *P. lima* and *G. catenatum* cells [49,50] modified the feeding behavior of *C. gigas*, showing a low filtration rate and the formation of pseudo-feces, mainly made up of intact *P. lima* cells, favoring the intake of *G. catenatum*.

On the other hand, laboratory studies have demonstrated that bivalves were capable of feeding on toxic phytoplankton, generating various effects. Pearce et al. [51] fed *C. gigas* (4 mm) with *P. rhathymum* under conditions that simulated harmful algal blooms (10^4^ cells/mL) for 21 days without recording mortality. Their results differ from those obtained in this investigation, where the death of 80% of the organisms exposed to 6 × 10^3^ cells/mL of *P. rhathymum* was observed with a direct relationship between the number of dinoflagellate cells and mortality. This relationship agrees with the results obtained by de las Heras [52] when *C. gigas* (3–5 mm) was exposed to this species. However, de las Heras [52] recorded 100% mortality in organisms exposed to 4.8 × 10^3^ and 6 × 10^3^ cells/mL and in the most concentrated extracts (20) of the three dinoflagellate species after 65 h exposure. The differences in mortality between de las Heras’ work [52] and the results in this study could be explained through the analysis of the organisms used. In this research, *C. gigas* spat in the bioassays were slightly larger (5–7 mm), which indirectly provides them with greater resistance to this type of exposure by decreasing the mass/mass ratio (dinoflagellate/oyster) or volume/mass (extract/oyster), which finally has a dilution effect. In addition to the aforementioned, the potential effects of different species of harmful algae and their toxins can vary in bivalves depending on a series of species-specific or individual responses [53].

The impact of phycotoxins in bivalves can be grouped into behavioral, pathological, genetic, genomic, or proteomic effects [54]. The study of proteomics effects in the early stages of development plays an important role because the proteomic changes that can occur in the early life stages of organisms emphasize the importance of their development and survival in changing environments, such as coastal environments [28].

The proteins involved in immune system were Toll-interacting protein isoform X4 (Tollip) and Cathepsin L1, identified in *C. gigas* exposed to whole cells. Tollip negatively controlled the Toll-like receptor (TLR) signaling pathways with which TLR-mediated activation of innate immunity controls not only host defense against pathogens but also immune disorders [55]. Based on this fact, the organisms exposed to *P. lima* and *P. minimum* by decreasing Tollip expression should cease to control the TLR receptors, experiencing an overactivation of the immune system—a mechanism reported by de las Heras [52] when an increase in hemocyte concentration was observed. On the other hand, the organisms exposed to *P. rhathymum* inhibit the signaling cascades activated by TLR when they showed Tollip upregulation. Thus, the immune system defenses do not work, making them prone to attacks by pathogens and causing organism death, whereas the presence of cathepsin L1 on bivalve mollusk has been demonstrated in previous studies and their expression pattern exhibited an increase after they were exposed to various treatments [56,57,58,59,60]. In this study, Ctsl1 was suppressed in the presence of *P. lima* and *P. minimum*, showing downregulation in the presence of *P. rhathymum*. This result indicates that Ctsl1 found in this study could have been involved in some defense mechanisms against pathogens or in response to non-own particle recognition. The suppression and downregulation of this protein indicates that exposure to organisms of the genus *Prorocentrum* could affect the first line of immune defense, leaving organisms vulnerable to pathogen attack, thereby causing death.

The proteins associated with the stress response identified in this study have been investigated very little in mollusks. Corporeau et al. [61] reported the downregulation of Dihydropteridine reductase in *C. gigas* caused by stress due to herpesvirus OsHV-1 infection, suggesting a possible effect on endothelial cells. This effect could occur in organisms challenged by whole cells of *P. lima* and *P. minimum*, where they did not counteract stress when downregulation was recorded, showing higher mortality (83.33%). The main studies carried out in periostin have reported that this protein is differentially expressed in various tissues. Luo et al. [62] reported downregulation in the gonads of male oysters *Crassostrea angulata* exposed to Bisphenol-A. Huan et al. [63] proposed that this protein was related to development and growth because the periostin sequences showed a different expression pattern among different larval stages of the clam *Meretrix meretrix*. Payton et al. [64] described an upregulation in the periostin genes when *C. gigas* was exposed to *Alexandrium minutum*—a species known to produce paralytic shellfish toxin and harmful algal blooms. The opposite effect was recorded in this study, where the proteomic analysis revealed a suppression when *C. gigas* was challenged by *P. lima* and *P. minimum* and a downregulation of this protein after exposure to *P. rhathymum*. Despite these studies, no direct evidence has been provided on the role of periostin in mollusks. However, these results suggest that it could be involved in some regulatory mechanism for environmental stress.

During *C. gigas* challenge by organisms and extracts of the genus *Prorocentrum*, the differentially expressed proteins identified and involved in muscle contraction and cytoskeleton morphology were Actin cytoplasmic, Myosin essential light chain, Tropomyosin isoform 2, and Troponin C. The expressions of these proteins suggest that biological processes, such as cytoskeletal activities, contractile cell functions, and muscle contraction, in oysters is affected, mainly in the abductor muscle where valve closure is affected. Similar results have been observed in previous studies in some bivalve species when challenged to different contaminants, where proteomic studies have reported the upregulation of actin, isoforms of tropomyosin, and light chain myosin on *Chamelea gallina* when exposed to Aroclor 1254 and copper(II) and the downregulation of actin in the presence of tributyltin and arsenic (III) exposure [32]. In the same way, Thompson et al. [65,66] found that proteins associated with cytoskeleton activity (actin, actin-2, tropomyosin, and myosin) were affected in *Saccostrea glomerata* by exposure to copper (Cu) and lead (Pb), and with the latter, tropomyosin concentration increased fourfold, affecting motility and cellular plasticity of hemocytes. Troponin C, the calcium receptor subunit [67], is still unclear in mollusks; some studies on TnC have been performed on *Patinopecten yessoensis* [68,69], *Ruditapes philippinarum* [70], and *C. gigas* [71], associating it with physiological functions, mainly in muscle tissues as an important part in muscle formation and contraction. However, studies carried out by Funabara et al. [72,73] in *Pinctada fucata* indicate that TnC genes are mainly expressed in adductor phasic muscles and rarely in adductor catch muscles, gills, mantles, and feet. The previous suggests that TnC may not have a role in catching muscle contraction; thus, the physiological roles of Tn in mollusks somehow may have undergone some divergence from vertebrates.

## 4. Conclusions

The dinoflagellate species *P. lima*, *P. minimum*, and *P. rhathymum* produce toxic compounds capable of causing mortalities and affectations at the proteome level in *C. gigas* spat, where exposure time was the factor that influenced mortality the most.

After 72 h of exposure, the biological functions of proteins affected by exposure to whole cells in *C. gigas* were the immune system, stress response, contractile systems, and cytoskeletal activities, while exposure to organic and aqueous extracts mainly affected proteins involved in muscle contraction and cytoskeleton morphology.

In general, this research demonstrates the applicability of proteomics in the study of oysters exposed to toxic organisms of *Prorocentrum* genus. Thus, the way these toxins act is mainly by affecting the expression of proteins that are responsible for the contractile systems.

The information generated in this study enriches the knowledge on early bivalve developmental stages. It also provides an approach for new bioassays and the generation of new, more specific analytical tools that allow for the main effects of algal proliferation on the early stages of bivalve mollusks to be monitored, characterized, and elucidated.

## 5. Materials and Methods

### 5.1. Biological Material 

The Japanese oyster *C. gigas*, 5–7 mm in shell length, were purchased from the “Acuacultura Robles SPR de RI” farm; upon arrival to the laboratory, the oysters were placed in an acclimation tank with natural filtered seawater to a temperature of 21 °C, salinity 34 UPS, and a light regime (12 h/12 h L/D) for five days. The oysters were fed with *I. galbana* microalgae (6 × 10^3^ cells/oyster/day).

The *Prorocentrum* species used in this study were obtained from the collection of marine dinoflagellates of CIBNOR S.C. (CODIMAR [74]). *P. lima* (PLHV-1) was grown in f/2 medium [75], while *P. rhathymum* (PXPV-1) and *P. minimum* (PIPV-1; currently regarded as a synonym of *Prorocentrum cordatum* (Ostenfeld) J.D. Dodge [7]) were grown in modified GSe medium [76]. The cultures were grown up to the exponential phase in 2L Erlenmeyer flasks under controlled conditions of temperature and illumination (24 ± 1 °C; photoperiod 12:12 h light–dark with cold white fluorescent lights (irradiance 150 µE m^−2^ s^−1^). Cell density was determined by Sedgewick-Rafter camera counts in a microscope (OLYMPUS BX43 optical, Olympus Corp., Tokyo, Japan) using 20× and 40× magnification.

### 5.2. Extract Preparation

The toxin extracts were prepared using a modification of the Yasumoto method [77]. In brief, 50 mL of each dinoflagellate culture was centrifuged at 1500× *g* at 4 °C for 10 min. The total cell concentrations of each species were *P. lima* 2.95 × 10^6^ cells, *P. rhathymum* 1.35 × 10^6^ cells, and *P. minimum* 9.4 × 10^6^ cells. The cell pellet was extracted with 10 volumes of 80% methanol; subsequently, a volume of dichloromethane was added. The obtained phases (aqueous, upper; organic, lower), were collected separately. All fractions were dried by vacuum distillation. The organic phase (OE) was dissolved in 5 mL of 1% Tween 60 (Sigma Chem. Co., St. Louis, MO, USA) and the aqueous phase (AE) in 5 mL of distilled water.

### 5.3. Exposure Experiments

All bioassays were performed in triplicate in 6-well plastic culture plates (BD Falcon Multi-well flat bottom sterile plates, Cat. 62406-155). Ten organisms of *C. gigas* were placed in each well, with 5 mL of diluted extracts or whole cells (depending on the case). On each cell, a complete treatment refill was carried out daily under identical conditions. Observations were made at 18, 42, 65, and 72 h of exposure; dead organisms were collected daily and stored at −80 °C until processing.

#### 5.3.1. Exposure of *C. gigas* to Organic (OE) and Aqueous (AE) Extracts

*C. gigas* 5–7 mm in length were exposed separately to organic (OE) and aqueous (AE) extracts of each dinoflagellate species at concentrations of 20, 10, and 5 ppt. The controls consisted of a 1:50 dilution of 1% Tween 60 for OE and a 1:50 dilution of distilled water for AE, both on sterile seawater.

#### 5.3.2. Exposure of *C. gigas* to Whole Live Cells of *Prorocentrum* spp.

The second experiment consisted of exposing/feeding oysters with whole cells of each tested species at densities of 1200 2400 3600 4800 and 6000 cells/mL. The control treatments consisted of 6000 cells/mL of fresh *I. galbana* culture.

### 5.4. Protein Extraction and Quantification

For protein extraction, ten individuals of *C. gigas* from each treatment were macerated (on ice bath) in 1.5 mL Eppendorf tubes with a glass pistil in 250 mL of cold phosphate buffer (4 °C; pH 7.8) containing 0.01% of a cocktail of protease inhibitors (PMSF SIGMA -ALDRICH, St. Louis, MO, USA) (in three replicates). After grinding for 5 min, samples were centrifuged at 8900 × *g* for 10 min at 4 °C. The supernatants obtained were recovered in new tubes, and total protein content was quantified by Lowry’s method [78]. Samples were kept at –80 °C until use for further analysis.

### 5.5. Proteomic Analysis

#### 5.5.1. Isoelectric Focusing (IEF, First Dimension)

For the proteomic analysis, first-dimensional electrophoresis was performed on immobilized pH gradient (IPG) strips with a pH gradient of 3–10 (ReadyStrip^®^IPG 7 cm; #163 2000, Bio-Rad Laboratories, Hercules, CA, USA) following the manufacturer’s instructions. First, 500 µg of protein was dissolved in 600 µL of rehydration buffer (urea 8M, CHAPS 2%, DTT 50 mM, Bio-Lyte^®^ Ampholites (Atlanta, GA, USA) 3–10 0.2%, and bromophenol blue 0.005% (*w*/*v*)). Then, the IPG strips were rehydrated at 20 °C for 14 h with 300 µL of the previous buffer–protein mixture. After rehydration, the strips were removed from the focus tray and the unabsorbed proteins were carefully removed. The IEF was performed using a PROTEAN^®^ IEF cell (Cat. 165-4000, Bio-Rad Laboratories, Hercules, CA, USA) according to the following IEF parameters: 20 min on a gradient up to 250 V-h, 120 min on a gradient up to 4000 V-h, and 160 min on a gradient up to 10,000 V-h until a total of 14,000 V-h was reached at 20 °C in 5 h. Along the complete run, the current was limited to 25 µA per strip, and temperature was 20 °C to avoid overheating and protein degradation. Finally, the gel strips were removed and stored at 80 °C until use for the second electrophoretic dimension.

#### 5.5.2. Sodium Dodecyl Sulfate Polyacrylamide Gel Electrophoresis (SDS-PAGE, Second Dimension)

After the first dimension, the IPG strips were equilibrated for 10 min (with gentle vertical shaking) on the equilibration buffer (urea 6 M, SDS 2% (*w*/*v*), Tris-HCl pH 8.8 0.375 M, glycerol 20% (*v*/*v*), and DTT 2% (*w*/*v*)). Then, a second buffer-equilibration was performed, but in this case, the DTT was replaced with iodoacetamide 2.5% (*w*/*v*) in the equilibrium buffer. Then, the IPG strip were placed on the polyacrylamide gel with an agarose overlay (12.5%). The electrophoresis was run on the Mini-protean II Cell (Cat. 165-2941, Bio-Rad Laboratories, Hercules, CA, USA) at a constant voltage of 100 V until the blue dye reached the bottom of the gel. The SDS-gels were stained for 3 h in a solution containing hydrated aluminum sulfate 5%, Coomassie Brilliant Blue G-250 0.2%, methanol 10%, and ortho-phosphoric acid 2% and de-stained using a solution containing 10% ethanol and 2% orthophosphoric acid.

#### 5.5.3. Image Analysis and Spot Detection

The two-dimensional gels were digitized by using a transparency scanner (UMAX PowerLook 2100XL, UMAX Technologies, Taiwan) with 16-bit depth and a resolution of 600 dpi. The images were aligned, and spots were detected and quantified with the Melanie7.0 software using the automated algorithm. All detected spots were carefully checked manually, removing artifact spots; thus, the analyses were limited to points that showed statistically significant variations in expression among the groups (ANOVA < 0.05) and absolute fold change (FC > 1.5).

#### 5.5.4. Identification of Differentially Expressed Proteins

After the differentially expressed proteins were detected (in quantitative terms), the selected proteins were manually picked from the gels and sent to the Laboratory of Biochemical and Instrumental Analysis of the Centro de Investigación y de Estudios Avanzados (CINVESTAV) del Instituto Politécnico Nacional (IPN) for MS analysis. The protein sequences obtained were identified by a BLAST-P search against the NCBInr sequence database, with an e-value cutoff of 1 × 10^−3^. The Kyoto encyclopedia of genes and genomes pathway database was used to identify in which metabolic pathways the differential expressed proteins were involved.

## Figures and Tables

**Table 1:** Mortality of *C. gigas* oyster spat exposed to whole cells (WC: 1200 2400 3600 4800 and 6000 cells/mL) and several concentrations of aqueous (AE) or organic (OE) *P. lima, P. minimum*, or *P. rhathymum* extracts.

Treatment		*P. lima*				*P. minimum*				*P. rhathymum*			
		Exposure Time (h)											
		18	42	65	72	18	42	65	72	18	42	65	72
**WC (cell/mL)**	Control	0	0	6.6 ± 0.6	3.3 ± 0.6	0	0	6.6 ± 0.6	3.3 ± 0.6	0	0	6.6 ± 0.6	3.3 ± 0.6
	1200	0	3.3 ± 0.6	53.3 ± 0.6	10.0	0	0	56.6 ± 1.3	16.6± 1.7	0	0	53.3 ± 0.6	13.3 ± 0.6
	2400	0	3.3 ± 0.6	53.3 ± 1.3	13.3 ± 0.6	0	0	66.6 ± 0.6	10.0	3.33 ± 0.6	0	56.6 ± 0.6	16.6 ± 0.6
	3600	0	6.6 ± 0.6	56.6 ± 0.6	10.0 ± 1.1	0	0	70.0	6.67 ± 0.6	3.33 ± 0.6	0	46.6 ± 1.7	20.0 ± 1.1
	4800	0	0	53.3 ± 0.6	23.3 ± 0.6	0	3.3 ± 0.6	56.6 ± 1.7	20.0 ± 1.1	0	13.3 ± 1.7	40.0 ± 1.1	23.3 ± 1.3
	6000	0	0	53.3 ± 0.6	23.3 ± 0.6	0	0	73.3 ± 1.3	10.0	0	3.3 ± 0.6	66.6 ± 0.6	10.0
**AE (ppt)**	Control	0	0	6.6 ± 1.3	0	0	0	6.6 ± 1.3	0	0	0	6.6 ± 1.3	0
	20	0	0	40.0 ± 1.1	40.0 ± 1.1	0	0	50.0 ± 1.1	23.3 ± 0.6	0	0	43.3 ± 0.6	43.3 ± 0.6
	10	0	0	30.0	46.6 ± 0.6	0	3.3 ± 0.6	30.0	33.3 ± 1.23	0	10.0 ± 1.1	53.3 ± 0.6	20.0 ± 2.2
	5	0	6.6 ± 0.6	20.0	33.3 ± 1.7	0	0	40.0 ± 1.1	10 ± 1.1	0	3.3 ± 0.6	46.6 ± 0.6	26.6 ± 0.6
**OE (ppt)**	Control	0	0	10.0 ± 1.1	0	0	0	0	10.0 ± 1.1	0	0	10.0 ± 1.1	0
	20	3.3 ± 0.6	6.6 ± 0.6	46.6 ± 0.6	20.0 ± 1.1	0	6.6 ± 3.33	50.0 ± 1.1	20.0 ± 1.1	0	13.3 ± 2.6	33.3 ± 0.6	30 ± 1.1
	10	3.3 ± 0.6	3.3 ± 0.6	53.3 ± 0.6	13.3 ± 0.6	0	3.3 ± 0.6	46.6 ± 1.3	20.0 ± 1.1	0	10.0 ± 1.9	43.3 ± 0.6	16.6± 1.7
	5	0	10.0 ± 1.1	43.3 ± 0.6	13.3 ± 0.6	0	0	36.6 ± 0.6	26.6 ± 0.6	0	6.6 ± 1.3	23.3 ± 0.6	36.6 ± 2.3

**Table 2:** Number of protein spots by treatment detected on total protein extracts obtained from *C. gigas* exposed to whole cells (WC), aqueous extract (AE), or organic extract (OE) of three *Prorocentrum* species. Resolution technique 2D-GE; software Melanie 7.0.

WC Treatment (434 Spots; 26 Regulated)	Species		
Type of Regulation	*P. lima*	*P. minimum*	*P. rhathymum*
Upregulated	14	10	13
Downregulated	3	5	4
Suppressed	3	5	3
Induced	3	1	2
**AE Treatment** (476 spots; 7 regulated)			
Upregulated	2	1	0
Downregulated	1	0	3
Suppressed	2	4	2
Induced	2	1	0
**OE Treatment** (623 spots; 13 regulated)			
Upregulated	1	3	3
Downregulated	4	6	4
Suppressed	6	3	3
Induced	1	2	1

**Table 3:** Identification of differentially expressed proteins in response to exposure to whole cells (6000 cells/mL) and aqueous (AE) or organic (OE) extracts, 20 parts per thousand (ppt) of *P. lima*, *P. minimum*, and *P. rhathymum*.

Spot ID	Protein	AAs ^1^	Query Cover ^2^	Protein Score ^3^	NP ^4^	Accession No ^5^	Expression Type ^6^		
							*Pl* ^1^	*Pm ^2^*	*Pr ^3^*
Whole Cells (WC)									
11	Toll-interacting protein isoform X4	281	14	62.4	2	XP_011451181.1	-	-	+
	Dihydropteridine reductase	237	15	60.8	2	XP_011421352.1			
14	Peptidase inhibitor 15-A isoform X1	325	57	53.4	4	XP_011454703.1	0	0	-
	Cathepsin L1	330	27	43.1	2	XP_011432380.1			
	Periostin	289	41	37	2	XP_011452718.1			
15	Tropomyosin isoform 2	284	99	111	14	NP_001295835.2	+	+	+
31	Myosin essential light chain, striated adductor muscle	157	94	62.4	4	XP_011411901.1	-	0	0
**Aqueous Extract (AE)**									
27	Actin cytoplasmic	535	85	139	8	XP_034314733.1	+	-	-
**Organic Extract (OE)**									
17	Troponin C isoform X2	150	100	36.2	2	XP_011429059.1	+	+	+
58	Retrograde protein of 51 kDa isoform X5	581	16	67.7	5	XP_019924109.1	1	1	1
	Mammalian ependymin-related protein 1	193	8	62	2	XP_011413901.1			
	Actin-2	376	26	57.8	4	XP_011444815.1			

^1^ Protein amino acid number. ^2^ The fraction of the query sequence that aligns to the subject sequence. ^3^ The blast score from the part of the subject sequence that aligns best to the query. ^4^ Number of matched peptides. ^5^ Accession number in the NCBInr database. ^6^ Protein expression type, + upregulated, − downregulated, 0 suppressed, and 1 induced.

## Data Availability

Not applicable.

## Supplementary Materials

The following are available online at https://www.mdpi.com/article/10.3390/toxins13070504/s1, Table S1: Summary of three-way ANOVA of *C. gigas* exposure to whole live cells of *Prorocentrum* spp., Table S2: Summary of three-way ANOVA of *C. gigas* exposure to the aqueous extract (OA) of *Prorocentrum* spp., Table S3: Summary of three-way ANOVA of *C. gigas* exposure to the organic extract (OE) of *Prorocentrum* spp., Table S4: Relative expression values of regulated protein spots that showed statistical differences (*p* < 0.005). Note: The processed data were obtained by subtracting the expression value of each spot with respect to the control value, Table S5: Peptide sequences of protein spots expressed differently and selected for identification by mass spectrometry.
