# Supplementary material for: Effect of Different Species of Prorocentrum Genus on the Japanese Oyster Crassostrea gigas Proteomic Profile
Source: Toxins (Basel). 2021 Jul 20;13(7):504. doi: 10.3390/toxins13070504 (PMC8310146; doi:10.3390/toxins13070504)
Supplement: Supplementary file 1 [file toxins-13-00504-s001.zip › toxins-1267767-supplementary.pdf]

Supplementary Materials: Effect of Different Species of *Prorocentrum* Genus on the Japanese Oyster *Crassostrea gigas* Proteomic Profile

Miguel Angel Matus Hernández and Norma Yolanda Hernández Saavedra

Table S1. Summary of three -way analysis of variance (ANOVA) of *Crassostrea gigas* exposure to whole live cells of *Prorocentrum* spp.

	Df	Sum Sq	Mean Sq	F value	Pr(>F)
Species	3	4.9	1.64	4.10	0.008135 **
Exposure time	3	921.4	307.13	765.818	<2e-16 ***
Cell concentration	4	1.6	0.41	1.025	0.396954
Species:Exposure time	9	55.3	6.14	15.319	<2e-16 ***
Species:Cell concentration	8	0.3	0.03	0.080	0.999640
Exposure time:Cell concentration	12	16.1	1.34	3.43	0.000299 ***
Species:Exposure time:Cell concentration	24	19.7	0.82	2.051	0.005696 **
Residuals	128	51.3	0.40		

Table S2. Summary of three-way analysis of variance (ANOVA) of *Crassostrea gigas* exposure to aqueous extract (AE) of *Prorocentrum* spp.

AE	Df	Sum Sq	Mean Sq	F value	Pr(>F)
Species	3	10.1	3.36	7.913	0.00011 ***
Exposure time	3	339	112.99	265.856	<2e-16 ***
Concentration	2	1.4	0.7	1.656	0.19742
Species:Exposure time	9	26.2	2.91	6.85	3.05e-07 ***
Species:Concentation	4	0.9	0.22	0.512	0.72707
Exposure time:Concentation	6	4.8	0.8	1.888	0.09296
Species:Exposure time:Concentation	12	22	1.83	4.317	2.96e-05 ***
Residuals	80	34	0.42		

Table S3. Summary of three-way analysis of variance (ANOVA) of *Crassostrea gigas* exposure to organic extract (OE) of *Prorocentrum* spp.

OE	Df	Sum Sq	Mean Sq	F value	Pr(>F)
Species	3	0.89	0.3	0.409	0.74718
Exposure time	3	293.7	97.9	135.034	<2e-16 ***
Concentration	2	1.39	0.69	0.958	0.388071
Species:Exposure time	9	25.24	2.8	3.869	0.000419 ***
Species:Concentration	4	0.06	0.01	0.019	0.999268
Exposure time:Concentation	6	11.5	1.92	2.644	0.021670 *
Species:Exposure time:Concentation	12	7.72	0.64	0.888	0.562656
Residuals	80	58	0.73		

Table S4. Relative expression values of regulated protein spots that showed statistical differences ($p < 0.005$) in *Crassostrea gigas* exposure to aqueous extract (AE) of *Prorocentrum* spp.

Spot ID	Control	*P. lima*	*P. minimum*	*P. rhathymum*	*P. lima*	*P. minimum*	*P. rhathymum*
	Original data				Processed data		
	**Whole Cells**						

**1**	1.138	1.166	1.120	0.856	0.028	-0.018	-0.282
**5**	1.212	3.966	5.003	2.739	2.754	3.792	1.528
**8**	0.716	1.262	1.518	1.345	0.546	0.802	0.629
**11**	1.149	0.808	0.314	1.178	-0.340	-0.835	0.029
**13**	1.117	1.130		1.586	0.013	0	0.468
**14**	1.433			1.247	0	0	-0.186
**15**	4.723	5.541	5.074	6.122	0.818	0.351	1.399
**16**	1.733	4.312	4.784	4.049	2.579	3.051	2.317
**21**	0.954	1.246	2.640	1.638	0.292	1.687	0.684
**25**	3.354	5.323	1.413	5.928	1.969	-1.941	2.574
**29**	1.938	7.362	7.053	6.715	5.424	5.115	4.777
**30**	1.650	2.264	2.239	3.007	0.613	0.589	1.357
**31**	2.134	0.332			-1.802	0	0
**35**	5.529	9.017	7.555	6.826	3.488	2.026	1.297
**36**	1.284	2.124	1.274	0.760	0.840	-0.010	-0.525
**37**	2.617	2.437	4.123	3.374	-0.180	1.506	0.756
**38**	2.027	3.594	2.547	2.791	1.567	0.520	0.764
**39**	3.213	5.233	2.226	2.802	2.020	-0.987	-0.411
**40**				1.283			1.283
**41**				1.409			1.409
**43**			1.906			1.906	
**44**		1.424			1.424		
**45**		1.238			1.238		
**46**		0.882			0.882		
**47**	1.718				0	0	0
**48**	1.411				0	0	0
				**Aqueous Extract**			
**21**	1.719			0.423	0	0	-1.297
**27**	5.823	6.338	5.576	4.471	0.515	-0.246	-1.351
**38**	1.710	1.260		1.228	-0.451	0	-0.483
**47**	1.646	1.911			0.265	0	0
**50**		0.992	1.996		0.992	1.996	
**55**		0.314			0.314		
**57**	1.542				0	0	0
				**Organic Extract**			
**9**	0.374			0.341	0	0	-0.033
**17**	0.624	0.682	0.650	0.816	0.059	0.027	0.193
**19**	0.306		1.256	0.700	0	0.950	0.394
**24**	4.380	0.903	1.975	2.576	-3.477	-2.405	-1.804
**29**	0.789			0.566	0	0	-0.222
**34**	0.891	0.294	0.159	0.560	-0.597	-0.732	-0.331
**39**	3.644	2.899	2.625	3.641	-0.745	-1.019	
**40**	1.222	0.613	1.079	1.857	-0.609	-0.143	0.635
**50**	1.115		1.400		0	0.285	0
**52**	1.884		1.531		0	-0.353	0
**58**		0.598	0.577	0.721	0.598	0.577	0.721
**70**			0.815			0.815	
**77**	4.207				0	0	0

Note: The processed data were obtained by subtracting the expression value of each spot with respect to the control value.

Table S5. Peptide sequences of protein spots expressed differently and selected for identification by mass spectrometry.

Spot ID	Peptide Sequence
11	YGFEQSTYYDLKVGISDPELFIPPRVGISDPELFIPPRYGFEQSTYYDLKVGISDPELFIPPRYGFEQSTYYDLKYGFEQSTYYDLKFLVYDETMQRFLVYDETMQRFLVYDETMQRIVTGQNPPSAGPMAEKIVTGQNPPSAGPMAEKIVTGQNPPSAGPMAEKLTEAMPFLLETKLTEAMPFLLETKKGNLGEMPSSGGDVFIHAKNMPANSVESFEDVYSFVYK**AQVMIGELPGDFLRVSYNQQQQMMADER**AQVMIGELPGDFLRLVTKEGNTALQNV**SLASKGSGLPANSSAVAILPVTLDTPMNR**
14	GNYISEYPYTKGPAcSKD**AWY**LAcFYDPKScHYTQIVWAK**ScHYTQIVWAK**GNYISEYPYTKScHYTQIVWAKScHYTQIVWA**KGNYISEYPYTKG**ENLAFDTNPKSVNIVVGQGVKTAERLSELGTLVKTAERLSELGTLV**KSVNIVVGQGVK**FNA**ANV**GATDSGFTDIK**FNAANVGATDSGFTDIK**NNcGIATSASYPTVNNcGIATSASYPTV
15	VYELEEQLSVVANNIKVYELEEQLSVVANNIKVYELEEQLSVVANNIKYKAISDELDQTFAELAGYVYELEEQLSVVANNIKTASEAEQEIQSLNRVLENLNNASEERTDVLEKVYELEEQLSVVANNIKTASEAEQEIQSLNRVYELEEQLSVVANNIKHSNLENEFDTVNEKHSNLENEFDTVNEKVLENLNNASEERTDVLEKYKAISDELDQTFAELAGYVLENLNNASEERTDVLEKYKAISDELDQTFAELAGYYKAISDELDQTFAELAGYYKAISDELDQTFAELAGYAISDELDQTFAELAGYYKAISDELDQTFAELAGYAISDELDQTFAELAGYLEAAEAKVYELEEQLSVVANNIKVLENLNNASEERTDVLEKVLENLNNASEERTDVLEKYKAISDELDQTFAELAGYVLENLNNASEERTDVLEKHSNLENEFDTVNEKYQECQTKYKAISDELDQTFAELAGYYKAISDELDQTFAELAGYVLENLNNASEERTDVLEKLQTATEKLEEASKYKAISDELDQTFAELAGYYKAISDELDQTFAELAGYYKAISDELDQTFAELAGYLQTATEKLEEASKAISDELDQTFAELAGYAISDELDQTFAELAGYVYELEEQLSVVANNIKVYELEEQLSVVANNIKLEAAEAKVYELEEQLSVVANNIKYKAISDELDQTFAELAGYYKAISDELDQTFAELAGYVYELEEQLSVVANNIKLQTATEKLEEAS**KHSNLENEFDTVNEKYQECQTK**VYELEEQLSVVANNIKHSNLENEFDTVNEKYKAISDELDQTFAELAGYVYE**LEE**QLSVVANNIKHSN**LEN**EFDTVNEKIQLLEEDMERSEERAISDELDQTFAELAGYVYELEEQLSVVANNI**KLAITEVD**LER**VYELEEQLSVVANNI**K**K**V**L**ENLNNASEERAISDELDQTFAELAGYLQTATEKLEEASKVYELEEQLSVVANNIKVLENLNNASEERTDVLE**KAISDELDQTFAELAGY**EVDRLEDELLAEKVLENLNNASEERLAITEVDLEREVDRLEDELLAEKKVLENLNNASEERVLENLNNASEERTLQVQNDQASQRKLAITEVDLERAKIEEDLTSLQKAISDELDQTFAELAGYLAITEVDLERAEARTLQVQNDQASQRIEEDLTSLQKKVLENLNNASEERAEQLEQQLRKLAITEVDLERIQLLEEDMERAEQLEQQLRYKAISDELDQTFAELAGYTASEAEQEIQSLNRLAITEVDLERAEARYKAISDELDQTFAELAGYTASEAEQEIQSLNRMEEAEKTASEAEQEIQSLNRVLENLNNASEERTDVLEKTASEAEQEIQSLNRVLENLNNASEERTDVLEKYKAISDELDQTFAELAGYVLENLNNASEERTDVLEKYKAISDELDQTFAELAGYYKAISDELDQTFAELAGYYKAISDELDQTFAELAGYAISDELDQTFAELAGYYKAISDELDQTFAELAGYAISDELDQTFAELAGYVLENLNNASEERTDVLEKVLENLNNASEERTDVLEKYKAISDELDQTFAELAGYVLENLNNASEERTDVLEKYKAISDELDQTFAELAGYYKAISDELDQTFAELAGYVLENLNNASEERTDVLEKLQTATEKLEEASKYKAISDELDQTFAELAGYYKAISDELDQTFAELAGYYKAISDELDQTFAELAGYLQTATEKLEEASKAISDELDQTFAELAGYAISDELDQTFAELAGYYKAISDELDQTFAELAGYYKAISDELDQTFAELAGYLQTATEKLEEASKYKAISDELDQTFAELAGYIQLLEEDMERSEERAISDELDQTFAELAGYLAITEVDLERKVLENLNNASEERAISDELDQTFAELAGYLQTATEKLEEASKVLELEEELKVVGNNMKVLENLNNASEERTDVLEKAISDELDQTFAELAGYEVDRLEDELLAEKVLENLNNASEERLAITEVDLERSLEISEQEASQREVDRLEDELLAEKKVLENLNNASEERVLENLNNASEERKLAITEVDLERSLEISEQEASQRAISDELDQTFAELAGYLAITEVDLERAEARVLENLNNASEERKLAITEVDLERIQLLEEDMERYKAISDELDQTFAELAGYTASEAEQEIQSLNRLAITEVDLERAEAR
31	NTDLQEDLEGNVKNTDLQEDLEGNVK**cVGLNPTLEIVR**NTDLQEDLEGNVKcVGLNP**TLE**IVRcVGLNPTLEIVRLTDDQVDEII**REGQGYISGAELRNTDLQEDLEGNVK**HLLSSLGERcVGLNPTLEIVR
27	DLYANTVLSGGSTMYPGIADRSYELPDGQVITIGNERDLYANTVLSGGSTMYPGIADRVAPEEHPVLLTEAPLNPKSYELPDGQVITIGNERSYELPDGQVITIGNERSYELPDGQVITIGNERSYELPDGQVITIGNERMQKEI

	TALAPSTMKMQKEITALAPSTMKVAPEEHPVLLTEAPLNPKVAPEEHPVLLTEAPLNPKSYELPDGQVITI<br>GNERSYELPDGQVITIGNERVAPEEHPVLLTEAPLNPKLCYVALDFEQEMGTAASSSSLEKMQKEITALAP<br>STMK**VAPEEHPVLLTEAPLNP**KVAPEEHPVLLTEAPLNPKD**LYA**NT**VL**SGGSTMYPGIAD**RDL**YANTVL<br>SGGSTMYPGIADR**SYELPDGQVITIGNER**MQKEITALAPSTMKMQKEITALAPSTMKMQKEITALAPSTM<br>KDLYANTVLSGGSTMYPGIADRVAPEEHPVLLTEAPLNPKQEYDESGPSIVHRMQKEITALAPSTMKMQK<br>EITALAPSTMKMQKEITALAPSTM**KSYELPDGQVITIGNER**EITALAPSTMKIKSYELPDGQVITIGNER**DL**<br>**YANTVLSGGSTM**Y**PGIADREITALAPSTMKIK**V**AP**EEHPVLLTEAPLNPKVAPEEHPVLLTEAPLNPKM<br>QKEITALAPSTMKSYELPDGQVITIGNERLDLAGRDLTDYLMKLDLAGRDLTDYLMKSYELPDGQVITIGN<br>ERSYELPDGQVITIGNERSYELPDGQVITIGNERMQKEITALAPSTMKGYSFTTTAERQEYDESGPSIVHRGY<br>SFTTTAEREIVREITALAPSTMKMQKEITALAPSTMK
17	SLALQINDEQLKSLALQINDEQLKSLALQINDEQL**KQLTDAHNTFNLFDK**
58	IIEEEAVGKQVDYYDVLYLHNVGKQVDYYDVLYLHNVGKQVDYYDVLYLHNVGKEYRFRIIEEEAVGKA<br>GRISYDEYAPRAGRISYDEYAPRFRIIEEEAVGKAGRISYDEYAPRFRIIEEEAVGKAGRISYDEYAPRFRIIEE<br>EAVGKFRIIEEEAVGKISYDEYAPRISYDEYAPRFRIIEEEAVGKFRIIEEEAVGKISYDEYAPRAGRISYDEYA<br>PRAGRISYDEYAPRISYDEYAPRISYDEYAPRISYDEYAPRIIEEEAVGKIIEEEAVGKIIEEEAVGKSYELPDG<br>QVITIGNERSYELPDGQVITIGNE**RVAPEEHPVLLTEAPLNPK**VAPEEHPVLLTEAPLNPKSYELPDGQVITI<br>GNERVAPEEHPVLLTEAPLNPKSYELPDGQVITIGNERSYELPDGQVITIGNERVAPEEHPVLLTEAPLNPK<br>VAPEEHPVLLTEAPLNPKVAPEEHPVLLTEAPLNP**KSYELPDGQVITIGNERLNDQIGEYEGEIATLR**LN<br>DQIGEYEGEIATL**RIGGLEDEVSKQRI**GGLEDEVSKQRLASEKECAELRIGGLEDEVSKQRRLQDDIAR**AQ**<br>**AVEEDLTFR**IGGLEDEVSKQRNVIDELSKEKLASEKECAELRSGSLDFFEVYNVLQKIFQSLDVDKNEKVG<br>EDFEEVDKDKNHTGPLSYEEFLKVGEDFEEVDKDKIFQSLDVDKNE**KYGFEQSTYYDLKVGISDPELFIPP**<br>**R**YGFEQSTYYDLKYGFEQSTYYDLKFLVYDETMQRILVATNLFGRGLAITFVSDETDAKGLAITFVSDETDA<br>KVVQLDYVENREAIFEELSEFTPGRR

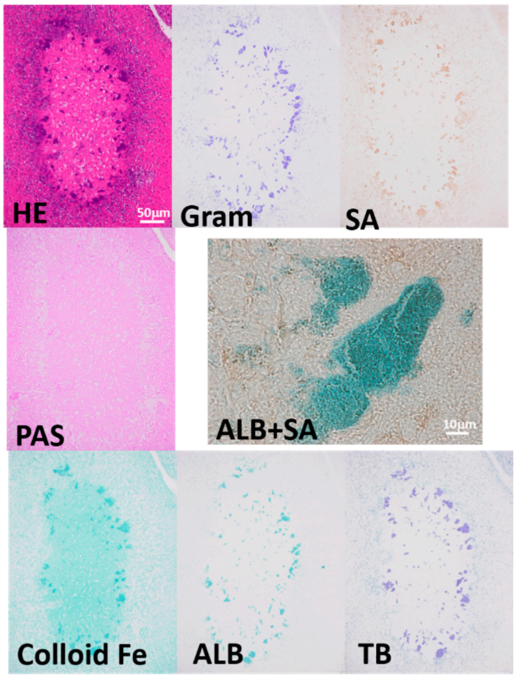

Figure S1. A large infected necrotic lesion in the liver of a mouse 10 days after OJ-1 injection. After injection of OJ-1 ×1 solution, some mice survived and their general condition was fine 10 days after bacterial injection; however, large extracellular necrotic lesions were sometimes developed in the liver. The lesion was examined for biofilm formation. The result was similar to the lesion 24 h after injection (Figure 1); many MRSA colonies developed around the necrotic focus, and they were accompanied with acidic polysaccharides detected by Colloidal Fe, ALB, and TB staining, but were negative for neutral polysaccharides (PAS). The double staining of ALB + SA showed a biofilm matrix containing acidic mucopolysaccharides.

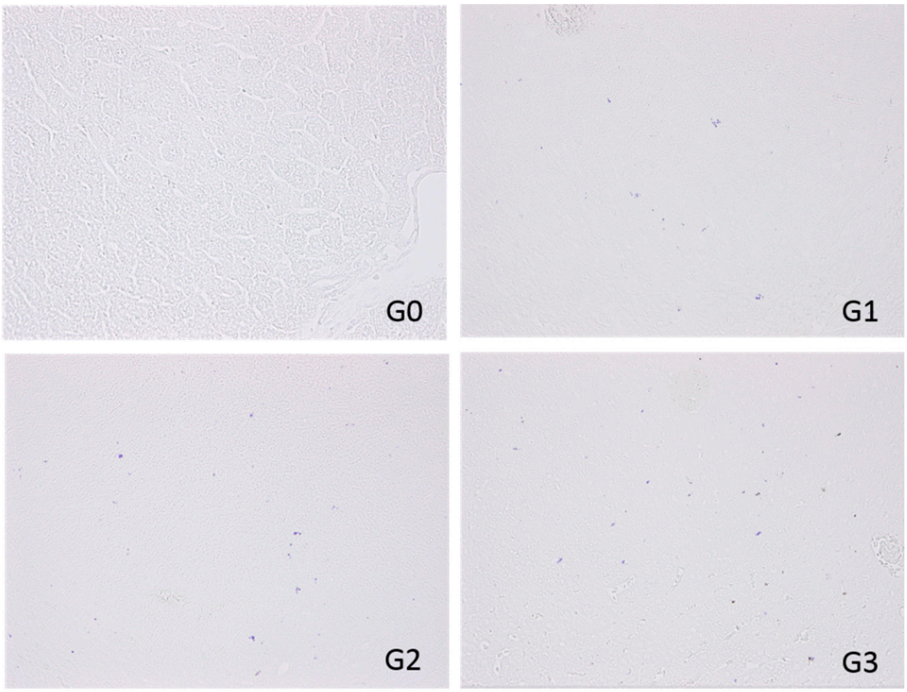

Figure S2. Reference picture for grading of gram-positive MRSA in the liver. Each liver picture was evaluated using a set of reference pictures for the grading of gram-positive MRSA.

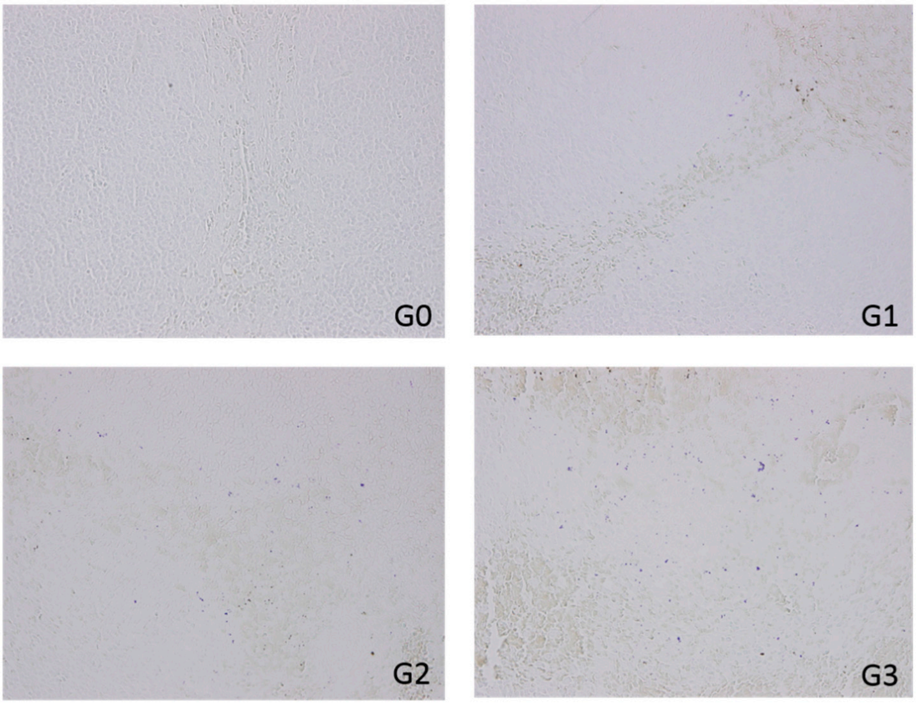

Figure S3. Reference picture for grading of gram-positive MRSA in the spleen. Each spleen picture was evaluated using a set of reference pictures for the grading of gram-positive MRSA.

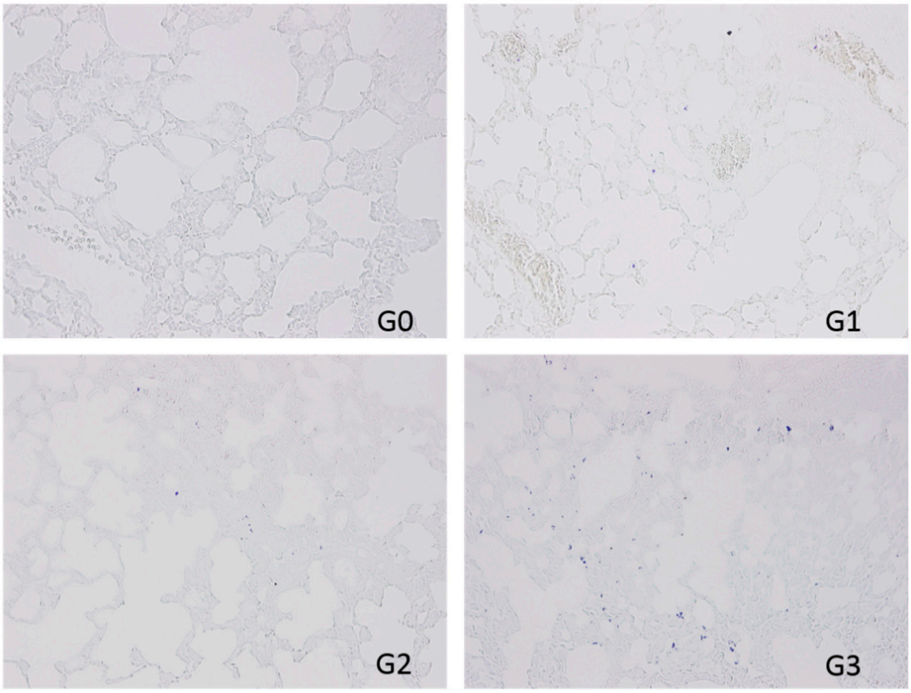

Figure S4. Reference picture for grading of gram-positive MRSA in the lung. Each lung picture was evaluated using a set of reference pictures for the grading of gram-positive MRSA.

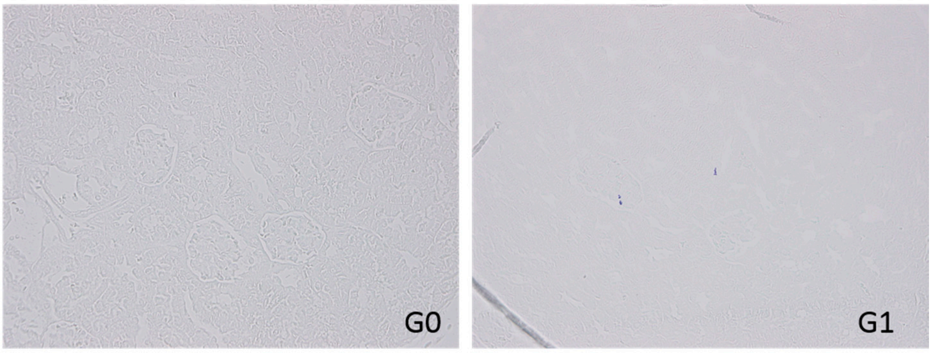

Figure S5. Reference picture for grading of gram-positive MRSA in the kidney. Each kidney picture was evaluated using a set of reference pictures for the grading of gram-positive MRSA.
